# What’s next for computational systems biology?

**DOI:** 10.3389/fsysb.2023.1250228

**Published:** 2023-09-19

**Authors:** Eberhard O. Voit, Ashti M. Shah, Daniel Olivença, Yoram Vodovotz

**Affiliations:** ^1^ Department of Biomedical Engineering, Georgia Institute of Technology and Emory University, Atlanta, GA, United States; ^2^ Center for Inflammation and Regeneration Modeling, University of Pittsburgh, Pittsburgh, PA, United States; ^3^ Center for Engineering Innovation, The University of Texas at Dallas, Richardson, TX, United States; ^4^ Department of Surgery, University of Pittsburgh, Pittsburgh, PA, United States

**Keywords:** data pipeline, digital twins, disease simulator, design principle, education, machine learning, systems medicine

## Abstract

Largely unknown just a few decades ago, computational systems biology is now a central methodology for biological and medical research. This amazing ascent raises the question of what the community should do next. The article outlines our personal vision for the future of computational systems biology, suggesting the need to address both mindsets and methodologies. We present this vision by focusing on current and anticipated research goals, the development of strong computational tools, likely prominent applications, education of the next-generation of scientists, and outreach to the public. In our opinion, two classes of broad research goals have emerged in recent years and will guide future efforts. The first goal targets computational models of increasing size and complexity, aimed at solving emerging health-related challenges, such as realistic whole-cell and organ models, disease simulators and digital twins, *in silico* clinical trials, and clinically translational applications in the context of therapeutic drug development. Such large models will also lead us toward solutions to pressing issues in agriculture and environmental sustainability, including sufficient food availability and life in changing habitats. The second goal is a deep understanding of the essence of system designs and strategies with which nature solves problems. This understanding will help us explain observed biological structures and guide forays into synthetic biological systems. Regarding effective methodologies, we suggest efforts toward automated data pipelines from raw biomedical data all the way to spatiotemporal mechanistic model. These will be supported by dynamic methods of statistics, machine learning, artificial intelligence and streamlined strategies of dynamic model design, striking a fine balance between modeling realistic complexity and abstracted simplicity. Finally, we suggest the need for a concerted, community-wide emphasis on effective education in systems biology, implemented as a combination of formal instruction and hands-on mentoring. The educational efforts should furthermore be extended toward the public through books, blogs, social media, and interactive networking opportunities, with the ultimate goal of training in state-of-the-art technology while recapturing the lost art of synthesis.

## Introduction

It’s tough to make predictions, especially about the future.

Yogi Berra (1925-2015)

Like so many other *bon mots*, this quote by the famous baseball player is almost a tautology, but it is particularly pithy because we have seen time and again how expert predictions have gone awry. Who would have predicted that junk DNA and futile cycles are not so junky and futile after all? Why are we not yet flying around with individual jet packs, as many science fiction writers prognosticated? And what happened to the prediction—made allegedly in the early 1950s by Thomas John Watson, the president of IBM—that the world would perhaps need a total of five computers, or by the Club of Rome that the world would run out of copper, given the rapid rise in telephone lines in the 1970s, before optical fibers had been invented?

Predictions regarding a new field of endeavor are especially treacherous. Over the past few decades, the parent disciplines of systems biology have moved with lightning speed from entire doctoral dissertations describing the sequencing of a single gene to microarrays with thousands of short DNA sequences, spotted by robots, and to large-scale gene editing with methods such as CRISPR-Cas9. In some sense, biology thereby started to become an “information science” (Baltimore and Denning, 2001). During the same time period, computing not only became many times more powerful than in the 1970s, but also much more widely available and accessible. Thus, any attempt to anticipate the global trajectory of systems biology is clearly fraught with risks. Then again, a few trends have been emerging that are likely to continue into the near future at least, and they are the topics of this perspective. It is impossible to portray all current and arising trends in experimental aspects of systems biology, and we will therefore focus in this article on the mathematical and computational aspects of systems biology, as well as on aspects related to the future of this field from the point of view of educating the next-generation.

Predicting the future of computational systems biology is further confounded by the fact that this scientific discipline does not yet have a generally accepted definition. Most working definitions include the terms “complexity” and “emergence” which, almost paradoxically, have no clear-cut definitions themselves. It is not difficult to list features of complexity, such as large numbers of components and processes, nonlinear interactions, feedbacks, threshold phenomena, and multi-scale operation, but a crisp definition is challenging. Similarly, it is often stated that systems can possess emergent properties, that is, properties that cannot be explained by examining system components in isolation, but the immediate question arises where these new features originate and how they materialize. How can we get something for nothing? Is emergence “illegitimate magic” (Bedau and Tomberlin, 1998; Voit, 2016)? The famous biochemist J.B.S. Haldane agreed with this view in 1932, when he wrote that “the doctrine of emergence … is radically opposed to the spirit of science” (Haldane, 1932). One should add that, 10 years later, Haldane expressed doubts that one could ever fully answer the question of what life is. Different thoughts on emergence have been proposed for engineered systems (Soria Zurita and Tumer, 2017), but the situation for biological systems is more complicated as we often do not even know all pertinent components and their interactions. The contemporary American philosopher Mark Bedau proposed as a partial solution to the dilemma the concept of “weak emergence,” according to which the high-level macro-state of a system has macro-properties that are determined and explained exclusively by interacting micro-states at lower levels and inputs from the environment. The emergence of new features can then be explained with computer simulations, although not by intuition (Bedau and Tomberlin, 1998).

At the very minimum, we can say that systems biology encompasses the assessment of as broad as possible a set of biological processes with a view toward synthesis and the extraction of novel insights. That definition is both aspirational and goal-oriented, and as such necessitates not only technological development but also a systems mindset that, in turn, requires a concerted effort encompassing three fundamental pillars: research, teaching and mentoring, and outreach to the world outside academic silos. Within each area, we discuss the current state as well as opportunities and challenges.

## Research goals

When discussing the future of a field, it is beneficial to revisit its current goals and, along the way, to take account of its status at the present time.

Intriguingly, computational systems biology has developed over the past two decades in a somewhat uncoordinated fashion, driven by research into a smorgasbord of topics. This almost uninhibited opportunity to advance in novel areas made the field very attractive (Androulakis, 2022). Beyond the application of systems biology to challenging problems in biology, medicine, agriculture, environmental stewardship and other areas of great societal importance, the goals of computational systems biology fall into three very broad categories, the first two of which, intriguingly, point in opposite directions. One thrust targets ever larger, more detailed models, whereas the other tries to strip as many distracting details as feasible from a system in order to focus on core features that simplify analysis without disrupting the system’s integrity, and to understand fundamental design and operating principles. The third goal is the efficacious and widespread translation of systems biology into solutions to challenging problems in biology and medicine. Below, we discuss the first two thrusts in greater detail and return to the third in the section on applications.

### Toward realism

The desire to increase the realism of models is not simply a matter of conceptually organizing large systems into well-integrated subsystems, but often entails severe computational challenges in terms of solving systems of equations and, particularly, instantiating the models with adequate parameter values. It will therefore be very beneficial, if not mandatory, to develop simplified surrogate models that retain the most pertinent information buried in highly detailed models.

Notwithstanding the technical issues, the pursuit of computational modeling of ever-larger systems is intuitively understandable, as the inclusion of more detail obviously has a good chance of making a model more realistic. This trend is logical, given the desire to obtain highly detailed, quantitative insights and predictions. It also creates an obvious paradox, namely, that, if taken to the extreme, the final product would be a 1:1-scale model that retains the full complexity of the original system whose intractable complexity triggered the need to generate a computational model in the first place. The situation is reminiscent of a short story by author J.L. Borges (Borges, 1946), who imagined an empire where the science of cartography became so exact that only a map on the same scale as the empire itself would suffice. An intermediate objective toward the lofty goal of a perfect one-to-one mapping between the real world and computational models has been the design of moderately complicated, reliable models of whole cells that permit interrogations at a much higher rate and at much lower costs than laboratory experiments. The prominent Japanese systems biologist Masaru Tomita called this goal of a reliable whole-cell model a “grand challenge for the 21st Century” (Tomita et al., 1999). Carrera and Covert (2015) explain the benefits of such a model as a tool for integrating heterogeneous datasets, identifying gaps in our knowledge, interpreting complex responses of cells, making predictions regarding different phenotypes of a cell, suggesting new experiments, and providing a safe and efficient framework for the design of genetically modified organisms with methods of synthetic biology.

As a first step in this direction, a model of arguably the simplest cell, the bacterium *Mycoplasma genitalium*, was assembled (Karr et al., 2012). Even though the task required enormous effort in terms of time and resources, the authors caution that the model is only a “first draft and that extensive effort is required before the model can be considered complete” (Karr et al., 2012). Since then, numerous scientists have expanded on these ideas and targeted other, more complicated cells. Of course, once reliable whole-cell models are feasible, the next targets will be models of tissues, organs, and even entire organisms, which will be discussed later in greater detail. Indeed, there is much excitement about “digital twins,” which are envisioned as computational analogs of individuals that will be so realistic that reliable experiments can be done computationally before the real-world individual is treated for a disease (Li et al., 2008; Shah et al., 2023). This aspect of the future is already underway, and various computational platforms have been proposed, including the Virtual Liver (Virtual_Liver, 2011), Virtual Brain (Virtual_Brain, 2023), Virtual Rat (University of Michigan, 2019), and ELIXIR (Martins dos Santos et al., 2022). The oldest of such efforts, the Physiome project (Physiome, 2011), has already generated very sophisticated models of the heart and other organs. These heart models are so accurate that they permit the recreation of myocardial infarctions and possible treatments.

At an even higher level of healthcare, the organization of hospitals, and healthcare in general, has long been compared to the complex organization of the human body—healthcare is composed of multiple pseudo-independently functioning teams of individuals, akin to organ systems of the body, who work together to triage, treat, and maintain homeostasis of the population, analogous to illnesses that affect individuals, at large. But what happens when an individual, hospital system, or nation is hit with a multi-system syndrome such as critical illness or COVID-19? These problems can be modeled in the same manner as a disease. Indeed, analogous to digital twins of COVID-19 patients (Day et al., 2021; Kim et al., 2021), digital twins of entire hospital systems have been leveraged during the COVID-19 pandemic to model how hospital capacity, stressed by patients ill with this disease, and patient vitals could affect a patient’s wellbeing in the hospital (Khan et al., 2022).

Whole-organism modeling platforms have by and large been applied to organisms relevant to human health and disease. Nonetheless, they are also important topics in agriculture and ecosystem management (Marshall-Colon et al., 2017). As specific examples, the Soybean Growth Simulation Model *SoySim* (University of Nebraska, 2022) simulates the growth of soy bean plants based on user-supplied input and makes management recommendations in terms of water use and fertilization. Another example is *WIMOVAC* (Windows Intuitive Model of Vegetation response to Atmosphere and Climate Change) (Song et al., 2017), which exploits biochemical and biophysical knowledge regarding photosynthesis to predict the responses of vegetation to environmental alterations or changes in light, temperature, CO_2_, humidity and other factors.

### Toward the quintessence of systems and a theory of biology

In stark contrast to increasingly larger models, the second major thrust of computational systems biology is to identify, characterize, and fully explain the most fundamental design and operating features governing biological systems (Mesarović et al., 2004; Savageau, 1985). Driving this pursuit is the assumption that nature has been testing, altering, and fine-tuning biological systems since their appearance 3.7 billion years ago. The optimization process is presumably not complete. However, biological systems we encounter today are, at least to some extent, better than alternative possibilities because evolution would probably have selected against particular designs if better alternatives had been available. The question of efficient—or possibly optimal—designs is not only of academic interest, but also offers strong guidance as we are beginning to create biomolecular systems and *de novo* organisms in synthetic biology, the sister field of systems biology (Bartley et al., 2017; Choi et al., 2019).

**FIGURE 1:**
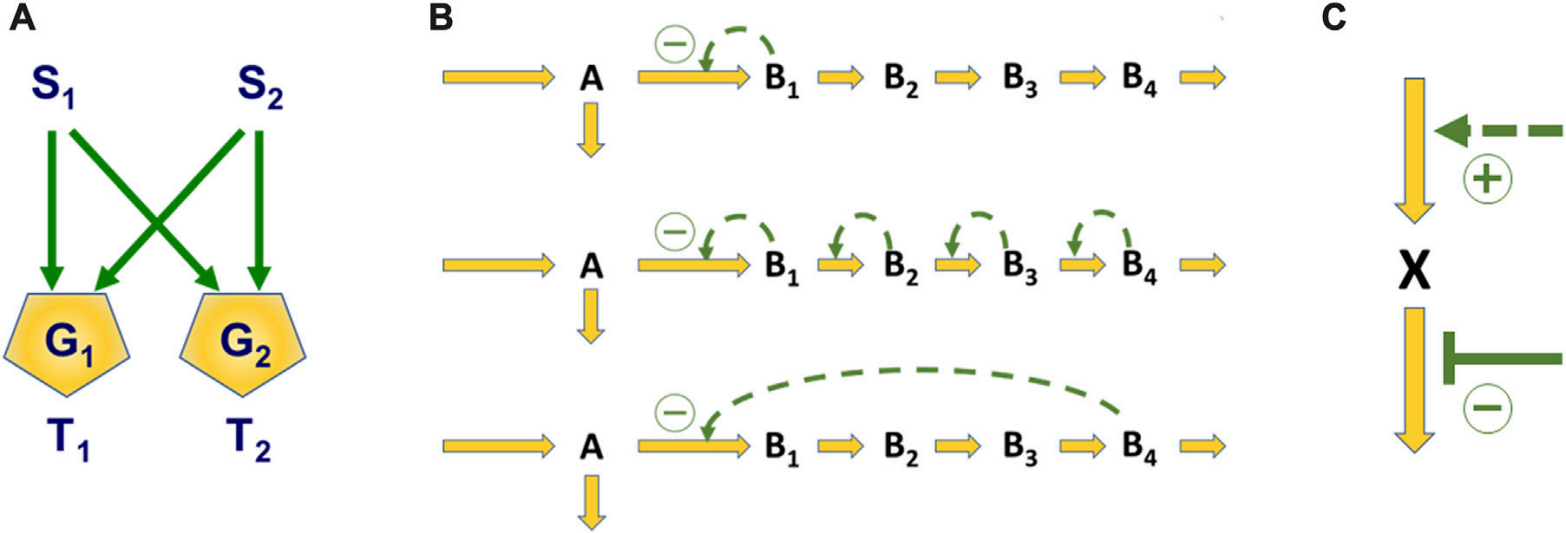
Illustrations of Questions of Design. Panel **(A)** shows a typical network motif in the form of a bifan, which integrates and transmits signals *S*_1_ and *S*_2_ to targets *T*_1_ and *T*_2_ according to rules governing the gates *G*_1_ and *G*_2_. Panel **(B)** displays three of many possible options for regulating the flux split at branchpoint A. In typical cases, the bottom design of feedback inhibition by the end-product is superior to other options. Panel **(C)** contains a simple input-output system where the question is whether an increase in production or a decrease in degradation is more effective if the demand for *X* is increased.

The search for optimal designs has involved the investigation of *motifs* (Alon, 2019), which are fundamental patterns or structures that we encounter time and again, efficacious regulatory patterns, and superior operating procedures. A typical motif is the so-called bifan, a signaling system that converts two inputs into two outputs; a generic illustration is shown in Figure 1A. The so-called *Boolean gates* G_1_ and G_2_ receive signals from S_1_ and S_2_ and transmit them (or not) to the targets T_1_ and T_2_ according to Boolean rules. For instance, if G_1_ is an AND-gate, it only passes on a signal if both S_1_ and S_2_ are firing. If G_2_ is an OR-gate, a signal is transmitted if either S_1_, S_2_, or both are firing. This type of motif can be found as the mechanism of “crosstalk” in numerous signal transduction systems.

A question of optimal design is illustrated in Figure 1B. Three linear pathways are shown, where material is leaving compound A at a branch point. Suppose the system’s role is to regulate its responses to changes in the demand for product B_4_ in the most efficacious manner. If so, what is the best manner of regulation? Many options are theoretically possible, but under most realistic circumstances, inhibition by the end-product can be proven to be optimal (Savageau, 1976; Alves and Savageau, 2000), and this is indeed a very common natural design.

Finally, consider the control of compound *X* in Figure 1C as an example for devising efficient operating strategies. If the demand for *X* increases from time to time, is it better to increase production (dashed arrow) or to decrease its degradation (blunted signal)? In this case, criteria outside the pathway dictate which strategy is better (Lee et al., 2011).

Explorations of motifs, design principles, and operating strategies are initial steps toward a deeper understanding of optimal functioning in biology. They are important as they inspire us to look for suitable building blocks of a theoretical foundation of biology. The Prussian philosopher and psychologist Kurt Lewin once said, “there is nothing so practical as a good theory” (Lewin, 1997: p. 288). To see the truth of this intriguing statement we must only look at physics. Its strong laws have made engineering possible and enabled the creation of machinery that functions well without a lot of trial and error. A stellar example is the exploration of space, where knowledge of physics has allowed us, for instance, to use a gravitational boost from Jupiter to let spacecraft like the twin Voyagers escape the Sun’s gravity on their way to Saturn, Uranus and Neptune (Mehta, 2018). Obviously, using Jupiter as a slingshot had never been tested experimentally, but the laws of physics stated that the strategy would work, and it did. It is hard to imagine what we could do with a similarly strong theory of biology, but it seems evident that the possible applications would be tremendous.

At this point, we are far from understanding biology sufficiently to formulate one or more theories. We don’t even know whether such theories might be crisp and deterministic, as in physics, or fuzzy in a sense that all statements would be probabilistic. It is also a question whether biological laws would be rather (or entirely) general or limited to small niches or subdisciplines. A first step to exploring potential theories is the systematic study of motifs and design patterns (Savageau, 1976; Savageau, 1985; Alon, 2019) as described above, along with the identification of recurring strategies with which nature has been solving problems for millions of years. One might think of the theory of evolution as an example (Voit, 2009). One could also point to the general principles of the genetic code, which is of enormous importance and by and large without exceptions. Without this “law,” we would have to start from scratch every time we wanted to determine the molecular machinery of a so-far unstudied species or even organism. A rather small portion of current research is trying to address issues of theories, and much more needs to be done.

While exploring potential theories, all our underlying assumptions must be examined carefully and objectively. For example, we presently do not know whether our current mathematics is appropriate and must ask whether other approaches would be better suited to handle, e.g., heterogeneous data or capture the essence of large-scale networks and systems. A pertinent, although tangential, issue is the expectation that any biological theories will likely be based on sophisticated mathematics and could therefore be quite intimidating to many traditional biologists. An early example in this pursuit is Chemical Reaction Network Theory [CRNT; (Horn and Jackson, 1972; Feinberg, 1987; Arceo et al., 2015)], which aims to identify properties of biochemical pathway systems independent of specific parameter values and thereby to discover design principles. As one might expect, CRNT is based on axioms and definitions that are formulated in an abstract mathematical manner and lead to theorems that follow from the axioms through sophisticated mathematical logic.

## Research tools and methodologies

To help coalesce data and modeling-based insights into theory, there is a need for the concomitant development of new tools, techniques, and methods. Experience has shown that new applications often spawn new tools and innovative tools make new applications possible. Both are often the consequence of blue-sky (“crazy”) ideas. Some emerging methodological research needs are sketched below.

### Data pipelines

Traditionally, experimental biologists were those who generated data and statisticians and mathematicians analyzed these data afterwards. The larger and more complicated the datasets have become, though, the more this division of labor has blurred. Even the generation and handling of raw data has become more complicated, as many experimental designs now involve robotic and computational support. This trend strongly suggests the benefits of creating semi-automated, carefully curated pipelines of analytical methods from raw data to static or dynamic models. These methods will likely hail from bioinformatics, statistics, mathematics, and computer science. Important tools at the intersection of data generation and analysis are machine learning (ML) and artificial intelligence (AI), which can greatly aid the extraction of core information from noisy datasets in an essentially unbiased manner. Along with such pipelines, it will eventually be beneficial to develop generally accepted standard operating procedures (“SOPs”).

A typical pipeline might contain the following phases.

1 Read typical raw data efficiently into a conveniently designed database
2 Use up-to-date methods for warehousing and accessing these databases
3 “Clean” the data by removing errors and flag perceived outliers, based on solid statistical principles
4 Extract significant information from the datasets, thereby distinguishing signal from noise
5 Convert simple and complex associations among the data into hypothesized chains of causes and effects, recognizing that feedbacks and other nonlinearities may feed effects back into causes
6 Create diagrams reflecting the hypothesized web of causes and effects
7 Convert these diagrams into symbolic models
8 Facilitate the choice of appropriate functional representations for these models
9 Infer values of model parameters from data
10 Implement the model with parameter values into computational structures and analyze these structures with analytical and simulation methods.

Some of these steps may appear to be straightforward but, in reality, pose significant technical challenges, with which the fields of bioinformatics and data science have been struggling for some while. For instance, the first step of depositing data may sound almost trivial but requires much thought, especially in the context of clinical data. In the case of proteomics by mass spectrometry, as another example, decisions regarding post-translational modifications, the numbers of peptides per protein and permitted missed cleavages, as well as numerous other aspects must be made before any measurement can be validly interpreted as novel information at the protein level. While numerous challenges remain, many tools useful for some phases of such pipelines have already been elaborated and utilized. An example is an effort by the European Research Infrastructure for Biological Data that aims to strengthen the infrastructure underlying systems biology (Martins dos Santos et al., 2022). Others are not so obvious; some are outlined below.

### From data toward models, using traditional statistics and machine learning

Rather than attempting to capture data directly with mechanistic models based on assumptions, which may or may not be correct, ML algorithms utilize clustering, segmentation, as well as stochastic and probabilistic architectures to predict outcome variables of interest directly from data (Angermueller et al., 2016). This type of modeling approach can either be supervised or unsupervised, depending on whether the architecture corrects itself with training based on the accuracy of the intended prediction or not, respectively (Angermueller et al., 2016). While ML algorithms are, in principle, apt for big datasets, many applications of ML in the field of systems biology have so far been hampered by overfitting, because they were applied inappropriately by violating the rule-of-thumb that a dataset must have roughly 10 times as many datapoints as there are independent variables (Chicco, 2017). This standard is quite problematic for any type of model in systems biology, as almost all data are sparse and corrupted by some level of noise. As an illustration, to use an ML algorithm responsibly, say in the prediction of survival or non-survival of patients with sepsis based on an assay of 20 different inflammatory mediators in the plasma, 200 patients worth of data would be necessary. While 200 patients may not seem like a lot, the necessary careful data collection would require substantial resources.

That said, there are challenges in systems biology for which ML algorithms are well suited. One application is the analysis of genomics data, which inherently qualify as “big-data,” given that they often assay hundreds of genes across multiple tissues and thousands of cells (Koumakis, 2020). However, if these thousands of cells come from a handful of patients (small sample size), as they often do, then ML algorithms for the analysis of -omics data should be used with caution (Koumakis, 2020). Even in large datasets, such as nationally aggregated data regarding the COVID-19 status of patients presenting to the emergency departments at four different hospitals in the United Kingdom, ML models performed best at predicting COVID-19 in patients when the parameters were tuned specifically to each hospital site (Yang et al., 2022). Lack of generalizability of trained ML algorithms and inadequately sized datasets, especially if they are sparse, make ML algorithms difficult to utilize in systems biology and particularly challenging to use in the context of developing computational models of human responses to disease.

### Dynamic hypergraphs

One solution to overcoming the sometimes limiting nature of ordinary differential equations (ODEs), which are typically used in mechanistic models (Edelstein-Keshet, 1988; Clermont et al., 2007), as well as the overfit models often generated via ML algorithms, lies at the intersection of basic science experimentation, traditional statistics, and dynamic and stochastic modeling. As an example, traditional experimentation targeting the *in vitro* and *in vivo* nature of pathologies provides granular data characterizing those components of the human body that lead to macroscopic outcomes such as fever, shock, and morbidity. Modeling macroscopic outcomes alone may oversimplify the state of the body, whereas modeling the cellular and molecular underpinnings of disease alone may lead to findings that are often irrelevant at the clinical scale. The need to augment *in vitro* data with *in vivo* data from both healthy and diseased animals and patient models across all systems biological projects will be critical as we trend toward developing digital twin models of pathologic states (Shah et al., 2023; Vodovotz, 2023). Traditional statistics will provide the core tools for rigorously testing the validity of experimentally inferred differences across time, patients, and datasets. Nonetheless, the utilization of ML algorithms will still be challenging as the size of the available datasets will often be much smaller than required based on the number of independent variables assessed.

**FIGURE 2:**
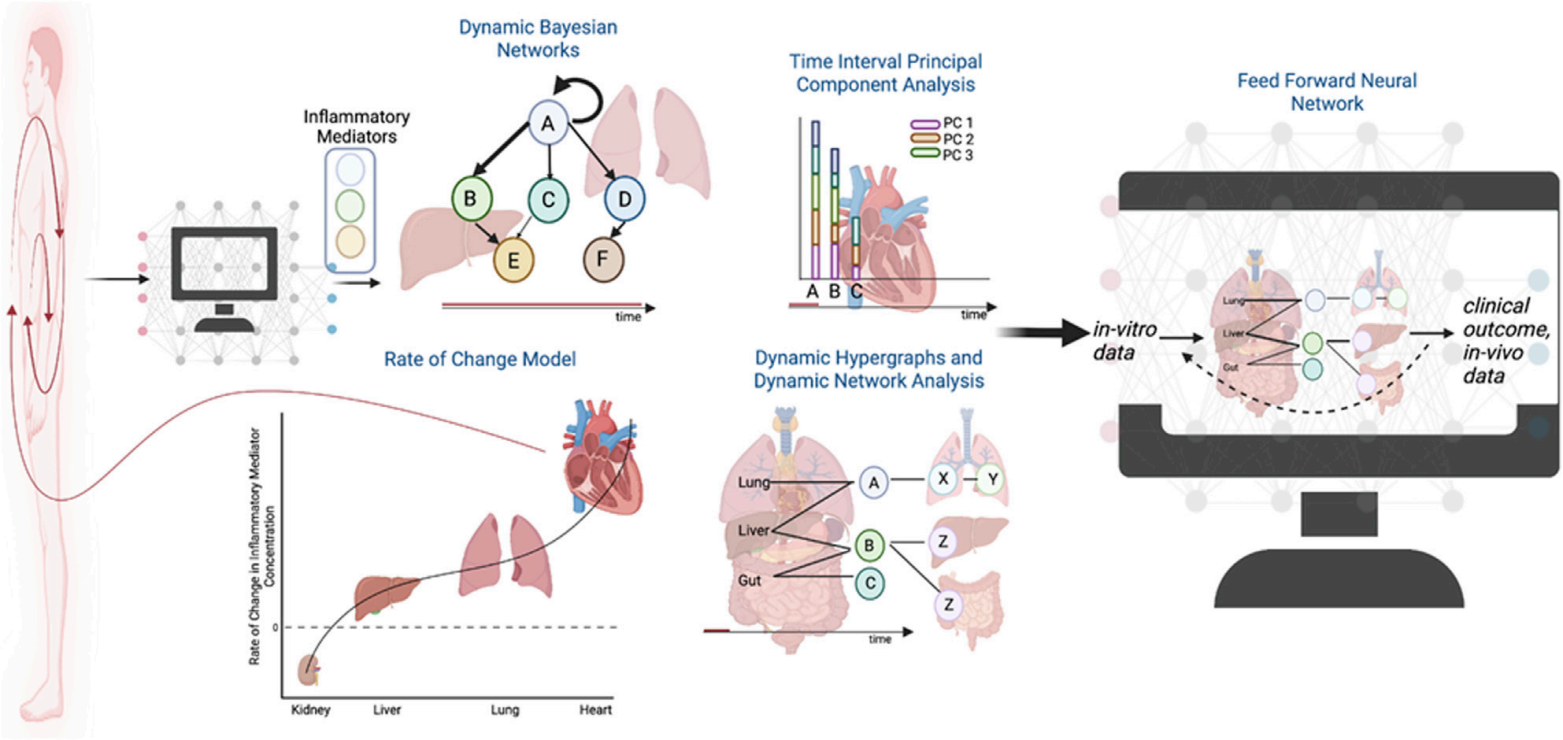
The future role of dynamic hypergraphs for a systemic assessment of disease. Rigorous statistics applied to *in vitro* and *in vivo* data can inform the development of ML architectures as the field trends towards the development of “digital twins” to model pathologic responses in the human body.

Dynamic hypergraphs, a permutation of the traditional hypergraph formalism, provide an opportunity to utilize both traditional statistics and rate-of-change models to interpolate the behavior of a biological system across tissues over time (Shah et al., 2022; Shah et al., 2023). Hypergraphs are defined by increased geometric flexibility in comparison to a traditional graph mode; namely, an edge in a hypergraph can connect any number of nodes (Schwob et al., 2019; Feng et al., 2021). As such, it is possible to define a multi-compartment model in which graph nodes represent tissue compartments (Shah et al., 2022). The edges can be defined in several ways and creatively leverage dynamic statistics. For instance, an edge can be defined by the correlation of an inflammatory mediator with itself across two time points, as the rate-of-change in expression of a gene within a tissue, by an ODE, or the correlation between two inflammatory mediators over time (Shah et al., 2023). Such models harness the strengths of traditional graph models and traditional statistics. Future extensions may seek to use the graph architecture suggested by hypergraphs as nodal structure for a feed-forward ML algorithm. By designing the ML algorithm, utilizing an architecture rooted in *vitro* data and traditional statistics, the predicted clinical outcomes of disease pathologies have a better chance of capturing the dynamics of a complex biological multi-compartment system (Figure 2).

### Harnessing the power of machine learning in computational systems biology

To explore potential generic advancements of ML and AI with respect to systems biology, it might be useful to branch out and explore fields in which ML and AI have been particularly successful in the recent past. One such field is the gaming industry, boosted by abundant resources and an estimated global worth of over $160 billion in the second quarter of 2021 (Bloom, 2021). Combined with an enormous public interest, a highly competitive ecosystem with accelerated evolution emerged within this field in a short period of time.

A key trend underlying the success of the gaming industry is the rise of game engines, which serve as toolboxes for game creation. These engines provide a comprehensive suite of development tools within an integrated development environment, enabling simplified and rapid game creation. Typical game engines include a rendering engine that enhances 2D models into 3D constructs, an engine creating physically realistic structures, scripting capabilities, sound integration, artificial intelligence, networking, streaming, memory management, threading, localization support, scene graphs, and video support for cinematics. Some examples of popular game engines are Unreal, Unity, Construct, Game Maker, and Godot (unreal_engine, 2023; Unity, 2023; Construct, 2023; GameMaker, 2023; Godot, 2023).

Applying similar concepts to the field of systems biology would address several challenges. Experimental biologists at present need to acquire numerous skills to conduct modern research, ranging from molecular chemistry and physics to statistics and computational sciences. It would greatly simplify their involvement in systems biology if they had access to a user-friendly, reliable set of tools for dealing with the technical aspects of computational modeling. In other words, the creation of an effective “bioengine,” analogous to game engines, could establish a standard for model construction and facilitate easier comparisons of models and their integration into more comprehensive, higher-level models. Such a standard could also serve as a reference point for the development of more effective future tools by providing creators with a baseline for performance comparisons. While some attempts have been made in this direction, such as SBML, (2023) and OpenWorm, (2023), they pale in comparison to what the gaming industry has achieved.

As an example illustrating some of the advantages of this strategy, consider the quest for designing a reliable whole-cell model. If an adequate physics engine were available that could accurately simulate the physics of a cell at the molecular scale, constructing a whole-cell model would become almost straightforward. One would start with the necessary raw materials and the DNA sequence of the investigated cell, create a transcription and translation process to convert the genetic information into proteins, and let the physics engine take its course. Obviously, the substantial challenge lies in developing such a physics engine. While this challenge appears to be overwhelming, one should note that progress has already been made toward its construction. For instance, AlphaFold (2023) is capable of predicting the 3D structure of proteins from their amino acid sequences with some reliability, thereby eliminating the need to simulate individual atoms. Furthermore, again gleaning successes of the gaming industry, game engines can already calculate the behavior of individual photons through ray tracing and animate characters with an astonishing resolution that allows intricate hair motion. Thus, by bringing together biologists, biophysicists, computer scientists, and game developers, fascinating possibilities could arise. Obviously, this type of effort would require significant funding, but the project is precisely of the type of high-risk, high-reward projects that are the mainstay of funding agencies such as the U.S. Defense Advanced Research Projects Agency (DARPA) or the newly established U.S. Advanced Research Projects Agency for Health (ARPA-H).

### Connecting snapshots through dynamic modeling

By nature, almost all experimental data consist of results obtained at one or several time points. In many cases, intuition is sufficient literally “to connect the dots,” but oftentimes intuition is overwhelmed, especially if the experimental findings are multi-dimensional, surprising, or even counterintuitive. One important role of mathematical and computational models in stems biology is the weaving of isolated results into cohesive trajectories that can explain how a system converts particular inputs into the outputs we observe. Ideally, the models capture the full dynamics of the system under the investigated conditions and are sophisticated enough to establish chains of causes and effects, which can be studied through detailed simulations. As an example, a fully dynamic model was able to explain how the bacterium *Lactococcus lactis* stops glycolysis just before environmental glucose, its substrate, runs out. This strategic stoppage is crucial for survival of the organism, as the downstream glycolytic intermediate phosphoenolpyruvate is needed for upstream glucose utilization (Voit et al., 2006; Dolatshahi et al., 2016a; Dolatshahi et al., 2016b).

Interestingly, even static models can yield new insights in both the static and dynamic nature of experimental observations. As an example, network modeling is now a fairly common means of visualizing cross-correlated data, with the underlying hypothesis being that such cross-correlations imply the presence of an organized biological program (Asthagiri and Lauffenburger, 2000; Neves and Iyengar, 2002; Sauro and Kholodenko, 2004; Janes and Yaffe, 2006). Most network modeling in the past has been static, *i.e.*, the data used to visualize the network were derived from a single time point or were treated as a single unit if they were comprised of multiple time points. When trying to explain a dynamic process, an initial step might be to create a “punctate” series of networks from data at individual time points, especially when the data available are sparse or comprised of only a few time points (Vodovotz et al., 2017; Sachdev et al., 2018). A conceptually similar but more sophisticated approach uses piecewise linearization of a dynamic process via assessment of cross-correlation across discrete time intervals (Mi et al., 2011). In the context of the inflammatory response, for example, methods such as Dynamic Network Analysis (DyNA) (Mi et al., 2011) were used to assess how inflammatory mediators are correlated with each other in individual tissue compartments over time (Vodovotz et al., 2008; Mi et al., 2011; Vodovotz and Billiar, 2013; Ziraldo et al., 2013; Namas et al., 2015; Zamora et al., 2021). This approach helped define novel, early mediators associated with hemorrhagic shock in experimental models of trauma (Mi et al., 2011) and mortality in the human context (Abboud et al., 2016), and also assisted in the definition of novel aspects of toll-like receptor 4-associated cross-tissue inflammation in experimental models of endotoxemia (Zamora et al., 2021; Zamora et al., 2023).

Dynamic Network Analysis is useful for defining, in a granular fashion, the time evolution of intercorrelated variables, but it does not highlight potential feedback nodes that might be relevant when thinking about how a complex system might be regulated. Topological Data Analysis and Dynamic Bayesian Network (DyBN) inference can address this deficiency by depicting time courses of variables as a network in which nodes are interconnected based on the algorithmically inferred likelihood that a given node impacts the appearance or decay of another (or the same) node (Grzegorczyk and Husmeier, 2011; Azhar et al., 2013a; Skaf and Laubenbacher, 2022). DyBN inference has been used to define potential feedback structures that regulate inflammatory programs (Namas et al., 2015; Azhar et al., 2021). In the context of blunt trauma, DyBN of circulating inflammatory mediators assessed within the first 24 h following severe injury suggested that HMGB1 and IL-23 exhibited positive self-feedback in non-survivors (Abboud et al., 2016). More recently, this method was used to infer the presence of a novel, chemokine-centered mechanism of inflammation control (Azhar et al., 2021).

Despite these advances in dynamic network inference using purely data-driven methods, the exploration of causality and regulation in complex systems still requires the construction of dynamic models. One challenge to their design is the fact that nature has not provided guidance regarding the most appropriate functional representations (Voit, 2017). However, powerful assistance to the envisioned automation of this step is provided by nonlinear canonical models, which are always constructed according to same rules, yet are flexible enough to permit capturing all relevant nonlinearities. Such canonical models prominently include Lotka-Volterra systems for straightforward analyses of interacting populations [e.g., (May 1973; Peschel and Mende, 1986; Voit and Savageau, 1986)] and power-law systems that are at the core of Biochemical Systems Theory [e.g., (Savageau and Voit, 1987; Savageau, 1996; Voit, 2013)].

## Applications

The scientific community and the public will ultimately judge the successes and failures of systems biology in terms of novel insights into the complexity of the living world and of applications to challenging problems. Examples of success stories of the past include, among many others, Eric Davidson’s work on regulatory networks governing body plan development (Peter and Davidson, 2011; Peter and Davidson, 2017), Denise Kirschner’s work on tuberculosis (Wigginton and Kirschner, 2001; Segovia-Juarez et al., 2004), and work by Gregory Stephanopoulos’ group on model-driven improvements of amino acid yield in *Corynebacterium glutamicum* approaching the theoretical limit (Vallino and Stephanopoulos, 1993). Also, we already mentioned model-based insights into inflammation and critical illness (An, 2004; Clermont et al., 2004; Vodovotz et al., 2008). It is to be expected that future applications of systems biology will often align with new trends in the experimental biomedical sciences and that they will address issues throughout the biological spectrum. Which of these applications will attract the attention of systems biologists is impossible to forecast. Nonetheless, some trends are emerging as potentially very powerful, as we discuss now.

### Translational systems biology and systems medicine

Many important applications will clearly fall into the general category of improving human health. An example will be a comprehensive collection of dynamic models of physiological systems that will not only be useful for education but also permit simulations of diseases and treatments for research questions. In fact, some realistic disease simulators already exist, and companies such as Entelos (Entelos, 2023), Immunetrics (Brown et al., 2015), Applied_Biomath (2023), and Nova_Discovery (2023) have for over two decades been developing realistic simulation platforms of utility to the pharmaceutical industry, which in turn uses these platforms to test the efficacy and safety profiles of drug candidates; many pharmaceutical and biotechnology companies now have internal groups carrying out this type of modeling as well. An exceptional example is an FDA approved simulator for insulin administration (Visentin et al., 2018), which can be used validly *in lieu* of experimental or clinical dosing studies.

More generically, new health-related fields are in the process of developing as spin-offs or extensions of systems biology. They come under the rubrics of Translational Systems Biology (Li et al., 2008; An, 2014), Systems Medicine (Apweiler et al., 2018; Wolkenhauer, 2021), Network Medicine (Chan and Loscalzo, 2012), Quantitative Systems Pharmacology (QSP) (Rogers et al., 2013) or similar terms and attempt to define means by which systems approaches might be used to improve all aspects of healthcare delivery, from disease models, to rational design of novel drugs or devices, to patient-specific models (digital twins), and ultimately to closed-loop, dynamic modulation of disease (Day et al., 2018; Vodovotz, 2023; Laubenbacher et al., 2022).

These applications of computational modeling grew out of the early days of systems biology, as enthusiasm for emerging data-driven and mechanistic modeling approaches to simple and simplified model systems began to gain acceptance. For example, the initial use of Principal Component Analysis to define cell signaling programs based on extensive time course data on cell signaling intermediates (Janes et al., 2005) led to applications regarding the inflammatory response in human disease states such as critical illness and acute liver failure (Azhar et al., 2013b; Namas et al., 2016; Schimunek et al., 2021). Early mechanistic modeling of the responses of inflammatory cells to infection (Alt and Lauffenburger, 1987) progressed to simulated clinical trials in the context of sepsis (An, 2004; Clermont et al., 2004). More generally, virtual clinical trials became accepted by regulatory agencies as beneficial, if not even necessary (FDA, 2011; Musuamba et al., 2021). Digital twin applications of mechanistic models in the context of inflammation followed thereafter (Li et al., 2008; Brown et al., 2015; Laubenbacher et al., 2022). In this regard, the field of QSP, which is aimed at drug development via disease models and virtual patients (Musante et al., 2002; Ekins et al., 2007; Allerheiligen, 2010; An et al., 2011; Iyengar et al., 2012; Visser et al., 2014; Klinke, 2015; Zhang et al., 2018), is a direct and important translational application of systems biology. Other specific applications of QSP target the translation of experimental or computational results from one species to another, in particular, from mice or rats to humans (Brubaker et al., 2020). The same goals have been pursued with physiologically based pharmacokinetic (PBPK) models for some while (Sager et al., 2015). As a recent example, preclinical data in swine were linked with clinical data from trauma patients via a 3-compartment ODE model of inflammation and coagulation. The model accurately predicted physiologic, inflammatory, and laboratory measures in both swine and patients, as well as predicting outcome and time of death in trauma patients. Furthermore, these studies suggested benefits of specific hemorrhagic shock resuscitation strategies (Cannon et al., in revision).

### Multi-scale models

The organization of biological systems as distinct but connected layers poses one of the grand challenges for biomathematical modeling, because processes occurring at the various layers often have different time scales and almost always focus on different types of variables. The higher layers usually correspond to a “big picture” of physiological events, whereas the lower levels account for increasing granularity and detail. When investigating a system at a high level, it is usually infeasible to carry along all details from lower levels, partly for technical reasons, but also because they would overwhelm insights at the higher level due to their sheer numbers and the fact that they typically run on much faster time scales. For instance, the growth of an embryo from a fertilized oocyte throughout the phases of blastula, gastrula, and further development may span several weeks, during which uncounted metabolic and cellular processes occur at a much more rapid pace. It has been argued [e.g., (Savageau, 1979; Kokotovic et al., 1980)] that the processes at lower levels are so fast that they are essentially always in a steady state, which permits their conversion from ordinary differential equation representations to explicit algebraic equations that may be entered as constraints in a much smaller set of governing equations. While separation of time scales is certainly an option for processes with time constants at different orders of magnitude, a sufficiently clear separation is not always feasible if the layers of biological organization are not all that distant. Consider two examples. The first is the clouding of the human lens, which constitutes an extremely slow, life-long process that is caused by clearly identifiable biochemical reactions occurring on an ongoing basis at the scale of parts of seconds (Ferreira et al., 2003). In this case, a timescale separation seems feasible, even though both processes occur simultaneously. Namely, on the fast scale, the responsible Maillard pathway is analyzed in sufficient detail, but only the overall accumulation of advanced glycation end products is retained for informing the slow-scale clouding process. In stark contrast, the heart beats with a frequency of approximately 1 Hz, which corresponds to a similar time scale as cardiomyocyte signaling. In this case, time-scale separation of the involved macro-physiological and molecular processes is problematic. Several reviews have outlined the challenges of modeling biological processes at multiple scales [e.g., (Schnell et al., 2007; Sloot and Hoekstra, 2009; Fletcher and Osborne, 2021)], but so far, no general, entirely satisfactory solutions have emerged.

One crucial aspect of multiscale modeling is the reliable information sharing among subsystems at different scales. As an illustration, suppose we could create an exceptional cell simulator, possibly with methods inspired by a game engine, as discussed before. How could we utilize knowledge from simulations at this subcellular level to infer events at the tissue or organ level? One approach could be the use of agent-based models at the lower scales if randomness is important for the behavior of individual cells or even smaller important modules (An, 2001; An, 2005; An, 2010; Glen et al., 2019). As we move to larger scales, grouping individual cells into aggregates could possibly allow application of the law of large numbers, which often permits valid model representation in the format of differential equations; as an alternative, one could again employ an agent-based approach with rules governing the behavior of these aggregates. At an even higher level, the aggregates could be merged into tissues, organoids, organs (Norfleet et al., 2020). A substantial challenge would still be the correspondence between levels, with an appropriate transfer of information. For instance, to move up to a higher level, information would have to be represented in a collective yet simplified manner, otherwise the higher-level models would be overwhelmed by detail. Similar issues would pertain to moving in the opposite direction (An, 2010). As a specific, practical example, moving down from an organ to an agent-based model focusing on its cells, should one initiate the latter all in the same state or in different states? A starting point might be a mean-field approximation, as it has been proposed (Kirschner et al., 2014), but much remains to be done. Indeed, the movement between scales will be a ubiquitous challenge for systems biology throughout the foreseeable future.

### Models of single-cell data

In the study of phenomena like cancer, it had long been assumed that, for instance, all breast cancers were more or less alike. This view had to be amended during the past two decades when it turned out that some breast cancers responded to some drugs, but other breast cancers, which appeared to be of the same type, did not. We now know that tumors and other cellular assemblies are often highly heterogeneous and that bulk experiments may yield misleading results (Di Carlo and Lee, 2006).

The possibility of studying single cells is particularly powerful for capturing rare events which, in the context of cancer, for example, may lead to metastasis. When studied in bulk, such events are often dismissed as noise or measurement error, although they may in truth contain very valuable hints regarding the organization and functioning of biomedical systems. Future work in this arena will require new methods for identifying and characterizing such rare events (see further comments in the *Conclusions*).

Current approaches to dealing with single-cell data often use methods of statistics, machine learning, and bioinformatics, but it is to be expected that systems biology will increasingly shed light on the dynamics of heterogeneous cell groupings. As a recent example, consider signaling processes during the epithelial mesenchymal transition (EMT), a process that drives the spread of tumors. EMT involves cells in morphologically and physiologically distinct states, and it had been assumed that the changes required different wiring modalities of the responsible signaling networks. However, a careful dynamic analysis of single-cell data demonstrated that rewiring is not necessary and that a constant network structure with minor parametric adaptations suffices to capture the signaling process throughout EMT (Wade et al., 2020). Another example is the extensive application of single-cell omics in the context of systems immunology (See et al., 2018). While many insights have been derived from this methodology, a key limitation is that the primary approach is that of pattern recognition and pattern-based classification based on clustering-based approaches such as *t*-distributed stochastic neighbor embedding (tSNE) and uniform manifold approximation and projection (UMAP) are extensions of older hierarchical clustering methods and are used to define the pattern of cells or molecules in any given experimental or clinical condition (See et al., 2018). As in the foregoing discussion of microbiome models, heterogeneity is a major challenge when attempting to glean mechanistic insights from pattern-based single-cell ‘omics data (An, 2014).

Dynamic statistical methods, including the dynamic hypergraphs mentioned before, are slowly emerging to address the heterogeneity of cell assemblies (Hasenauer et al., 2014; Loos et al., 2018; Wade, 2020; Vodovotz, 2023), but the field is still wide open for advancements.

### Models of microbiota

All organisms live in communities, and while macroscopic populations have been studied for a long time, recent interest has been shifting to mixed microbial communities, microbiota, or microbiomes, as they are alternatively referred to. Of direct importance for human health are microbiomes in and on our bodies, which are all different, depending on their location. Even our mouth microbiome varies in different niches (Deo and Deshmukh, 2019). The mammalian gut microbiome has received the most attention, given that it is critical for healthy digestion and metabolic wellbeing, but also for mental health, with dysfunction possibly leading to depression (Shoubridge et al., 2022). It has been estimated that our body is home to approximately 40 trillion bacteria (Sender et al., 2016).

Outside human health, microbial communities are directly associated with the health of ecosystems. A single Gram of soil can contain enough microbial DNA to stretch nearly 1,000 miles (Trevors, 2010). Lakes and rivers are often inhabited by ten to twenty thousand operational taxonomic units (OTUs), which cleanse the water of many types of pollutants (Dam et al., 2016; Callieri and Fath, 2019). Due to the enormous numbers of participants, their often-large diversity of species, and the fact that they can change dramatically over time, microbial communities are too complicated to permit intuitive assessments and are therefore an important application for computational systems biology. The specific quest is usually to characterize the interactions among different OTUs and to predict their future dynamics. The task has been approached in the past with network analyses or dynamic models. In the former case, correlation networks are constructed based on the presence, absence, or abundance of different species across multiple locations or time points (Ruan et al., 2006; Barberan et al., 2012; Faust et al., 2012; Friedman and Alm, 2012; Gilbert et al., 2012). More complex relationships have been derived from rule-based networks or regression analysis (Chaffron et al., 2010; Faust and Raes, 2012). Static correlation networks are well suited to address large and complex communities of thousands of species (Chaffron et al., 2010; Barberan et al., 2012), but they typically ignore the asymmetry of relationships between species and do not capture dynamic trends, which require dynamic systems models (Mounier et al., 2008; Stein et al., 2013; Berry and Widder, 2014; Marino et al., 2014; Dam et al., 2020). The inference of interactions for these dynamic systems is the subject of ongoing investigation (Davis et al., 2022; Olivença et al., 2022).

Other key challenges that remain for microbiome analysis center on the integration of proteomic and metabolomic data that could permit inferences beyond the simple distinction between the presence or absence of a given taxon. Additional goals include a better grasp of the large heterogeneity in microbiome composition among individuals, the impact of phenomena like circadian rhythms, and factors impacting microbiome dynamics in individuals and populations (Gilbert et al., 2018). Finally, a new approach to analyzing microbiomes that presents great potential is metagenomic enzyme discovery, which attempts to predict biocatalytic function directly from sequencing data. Although this strategy is still quite challenging to implement, some efforts are on their way (Robinson et al., 2021; Jia et al., 2022).

### Other microbial applications

Modeling microbial systems has become important in synthetic biology and metabolic engineering. Systems biology can be considered the theoretical framework for synthetic biology, and the two fields will probably share many applications in the future (Hanczyc, 2020; Ezzamouri et al., 2021). Most of the past and current efforts of synthetic biology have been focusing on microbial models, due to their relative simplicity, and this preference will most likely dominate the future, although mammalian stem cells have gained much interest in recent times. Similarly, computational models addressing microbial pathway systems will eventually become the standard for rational metabolic engineering (Alvarez-Vasquez et al., 2000; Harder et al., 2016; Gudmundsson and Nogales, 2021; Palsson et al., 2021). Applications in this field are manifold. Just one example is the manipulation of hydrogen production by bacteria, based on computational models and the manipulation of their molecular inventory. Promising approaches are direct mutagenesis and high-throughput screening assays (King et al., 2022), as well as the generation of hydrogen in a cell-free system (Hodgman and Jewett, 2012). These approaches are expected to gain from computational modeling support. Even more intriguing, oceanic cyanobacteria of the genus *Synechococcus* fix carbon and convert it into sugar within spatially well-placed internal structures, called carboxysomes, and the introduction of mutations of a gene responsible for the organization of the carboxysome could lead to higher photosynthesis and, subsequently, hydrogen (Savage et al., 2010).

More broadly, society must explore different stable states of coexistence between humans and complex microbial communities, on earth as well as possibly on other planets. These explorations, both experimental and model-based, must address not only environmental issues and food production, but also parasites. Beyond the well-known challenges of understanding host-pathogen interactions (Gutierrez et al., 2015; Garcia et al., 2008), the recent pandemic has implicitly suggested yet another application of systems biology to microbial problems. Namely, it will be more difficult in the future to obtain funding for gain-of-function research, due to its inherent danger (Board-of-Life-Sciences, 2015), which has become abundantly evident during the pandemic. A potentially powerful alternative to experimental studies might eventually consist of realistic simulations based on tools of computational systems biology.

Both research into new methodologies and theoretical advances and the application of these methods to challenging real-world problems require the involvement of new cohorts of students and postdoctoral fellows. Some of these may come from any of the parent disciplines of systems biology, but there are so many genuine features of the field that the education of new generations of scientists must be a high priority for our community. We will describe some of the needs and challenges of this aspect of systems biology next.

### Education

Before systems biology became accepted and appreciated, the dominant paradigm of biological research was reductionism. In coarse terms, this approach means that to understand an organism, we need to understand its organs; to understand these, we need to understand the tissues in the organ; to understand these, we need to understand the cells in these tissues, and so on, thus mandating a chain of investigations down to the basic building blocks of life. This approach has been tremendously successful, and we now have comprehensive molecular inventories of cells. While systems biology absolutely needs the knowledge derived from this *modus operandi*, it has become clear that reductionism is not entirely sufficient and that we need tools to reassemble the pieces into functioning entities. Indeed, this integration is at the heart of systems biology, and one significant achievement of systems biologists has been their emphasis on convincing traditional biologists to look at nature through the lens of connectedness and systemic regulation.

Given the importance of this new world view, what exactly do we need to teach and at what level? It is clear that an often-reinforced emphasis must be on core concepts such as complexity, dynamics, regulation, nonlinearities, threshold effects, and emergence. But what will be the most effective manner of conveying these concepts within relevant contexts? The educational community within systems biology has been struggling with these questions, without having found a perfect answer. The reason is that the genuine interdisciplinarity of systems biology creates four unique challenges for teaching and for the development of academic curricula (Voit and Kemp, 2011). First, it requires successful students to gain diverse skills from data generation to computational system manipulation, which entails at least basic knowledge of the core concepts of the biological sciences, chemistry, physics, computing, mathematics, and engineering, as well as mastery of a wide span of techniques. It is quite evident that no student can acquire as much knowledge in all these fields as a student concentrating on any one of them, which implies the need for careful consideration of which topics are truly necessary for understanding the inner workings of a biological systems. The second, associated challenge is the diversity of backgrounds and academic experiences among students possibly interested in systems biology. Some will hail from more traditional biology, while others may have backgrounds in mathematics, physics, or computing, with corresponding deficiencies in areas outside their major fields of study. The third challenge is the intrinsic complexity of biomedical phenomena, which is genuinely difficult to comprehend and even more difficult to teach. One aspect of this complexity is the often-cited emergence of systems behaviors that cannot be explained in terms of any of the components in isolation. While practitioners are well aware of examples of emergence, crisp definitions and conclusive explanations do not really exist. To put it bluntly, two core concepts of the new field of systems biology, namely, complexity and emergence, elude simple explanations, which creates a rather difficult starting point for education in the field. The fourth challenge is that many biologists and engineers are convinced that learning occurs best in a research lab, while coursework should be minimized. It seems that students retain knowledge better through hands-on learning, but it also appears that some formal classroom instruction is needed.

Educational programs in systems biology may take distinctly different formats. The lowest level of effort is the creation of a module within an introductory biology class. As an example, such a module was created on the topic of homeostasis, exemplified with the regulation of oxygen in the blood stream, and successfully implemented it in a first-year biology course at Spelman College (Ayalew et al., 2022). The same topic will be revisited in introductory math and computer science classes at Spelman, with domain-specific spins. For instance, the math module will look at the stability of steady states, the mathematical formulation of feedback inhibition, and other mathematical aspects, while the computer science module will focus on model coding, numerical methods for solving differential equations, and means of visualization. It is obvious that such cross-disciplinary training requires genuine buy-in from all instructors, as well as close coordination.

The creation of modules within existing classes exposes students to systems thinking in a very important, but minimal fashion. The next higher level of exposure is achieved with a semester-long course dedicated to so-called STEM (science, technology, engineering, and math) concepts. Ideally, such a course lets students experience the art of systems analysis and simulation with hands-on computer exercises. Outside the acquisition of techniques, a goal of this approach is to instill in students a “feel” for systems and their dynamics (Voit et al., 2012). Because systems biology is applicable to numerous problem spaces, it offers the opportunity to custom tailor a course to a specific topic or home department. Examples include a course dedicated to analyzing the systemic nature of a specific disease of interest (Voit et al., 2012) and a systems biology course with emphasis on quantitative systems pharmacology (Androulakis, 2022).

A typical critique of this educational strategy is that the computational exercises are necessarily relatively simple “sand-box problems” that lack relevance for the complicated, messy real world. While that might be so, one should remember that we learn geometry on the basis of ideal circles, triangles and perfectly straight lines, which do not exist in actuality, with the exception of construed examples.

**FIGURE 3:**
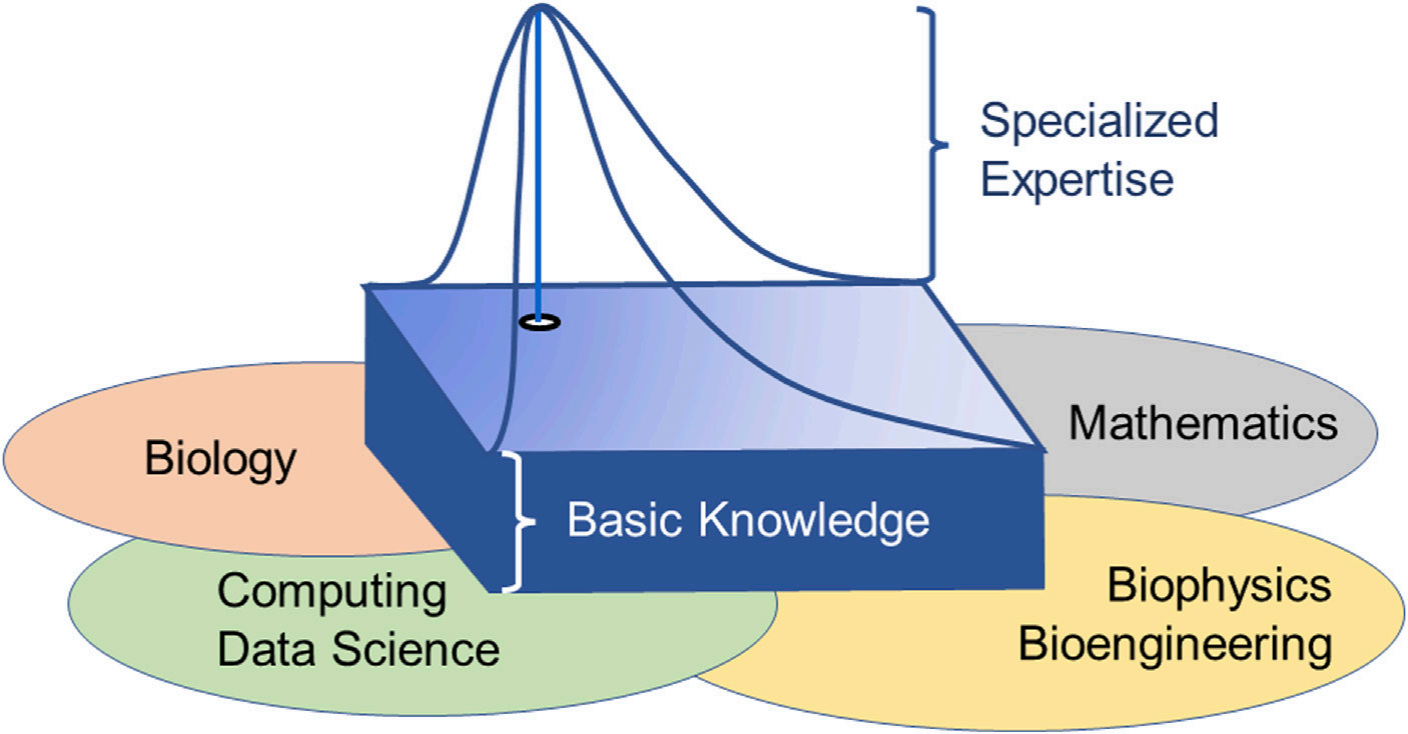
Educational target of achieving broad interdisciplinary knowledge combined with specific expertise in one niche. Because systems biology spans several disciplines, every student must acquire basic knowledge and expertise in the parent disciplines. At the same time, it is mandatory that each student becomes an expert in some limited area within the field.

Even a semester-long course can only address the tip of the iceberg. A more comprehensive experience with biological systems is obviously gained through an entire program that touches on fields tangential to biology and mathematics, such as computer coding, bioinformatics, data science, and possibly biophysics, and integrates knowledge from these fields into systems approaches. In the end, such a program must address the necessary basics of the parent disciplines but, at the same time, allow the student to gain true expertise in one subspecialty, both through classes and projects, as well as a thesis or dissertation (Figure 3).

In addition to strictly technical skills, aspiring systems biologists should be made aware that the scientific method underlying the new field is different from the typical method of posing, testing, and accepting or refuting hypotheses. Namely, the scientific method of systems biology spans two distinct worlds: reality and the domain of mathematics (Voit, 2019). In the process of using models to analyze biological systems, a piece of reality is transferred to the mathematical domain, where all analyses are executed, and the results are subsequently transferred back to the world of reality. The act of transferring information between the two worlds requires specific correspondence rules that govern simplification, abstraction, and mathematical formulation in the beginning of the modeling effort and the translation and interpretation of results, for example, from eigenvalues to biologically understandable concepts like stability, at the end.

As an important part of teaching the students about this conceptual difference, the students need to realize that different disciplines use their own languages, which must be translated in order to communicate among practitioners from the different fields (Voit, 2016). The necessary skills for this aspect may be learned by involving the students in consulting projects where, for instance, experimental biologists are aided in the development of a supporting model. It is also necessary to train students in the art of collaboration in large, multidisciplinary projects that might span several institutions with different scientific cultures and languages. Furthermore, aspiring systems biologists need to be made aware of the rapid evolution of the field of biology in general. For example, the nature of biological data is constantly changing, thus requiring systems biologists to become familiar with a spectrum of new technologies. Not long ago, microarrays were the frontier of genetic research, but they have by now been made almost obsolete by next-generation sequencing (Slatko et al., 2018).

Traditional teaching methods will continue to be important, but one should also mention new methods like notebooks such as Jupyter, Google Colab or Noteable. These applications facilitate the interpretation of code and explain text and program output, which decreases the user’s effort to understand the details of computer code. Of particular note, Open AI made a ChatGPT plug-in available in July 2023 that can read files and analyze them by running code (Franzen, 2023). This facility allows users to analyze data, create charts and data visualizations, perform math, create interactive HTML files, clean datasets, and perform other tasks, thereby greatly decreasing the effort to perform data analysis and enabling individuals with rudimentary training to engage in the interpretation of new information. It is not hard to imagine that similar developments in the field of systems biology might follow soon. If so, the new trend will catalyze a shift in the teaching of systems biology and change the present focus on technical skills to a more conceptual emphasis on the possibilities the tools of system biology allow.

Whichever format of formal education in systems biology is chosen, it will greatly benefit from the support of strong textbooks. Although the field is still young, it is a sign of its importance that several introductory books have entered the market in recent years. Examples include (Edelstein-Keshet, 1988; Kaneko, 2006; Sauter and Albrecht, 2012; Kremling, 2013; Robeva, 2015; Klipp et al., 2016; Voit, 2017; Alon, 2019). These books are designed to support classroom education but could also be used for self-learning by students as well as the interested public. Indeed, the latter group should not be underestimated as it is of great importance, as we will discuss next.

### Outreach

Systems biology has matured to a point where it is widely accepted among biologists. While this acceptance is without doubt a major achievement, we cannot rest but must share our excitement about the new field with the outside world (Voit, 2022). Over the time horizon of the next decade or two, this publicity will not be a luxury but an absolute necessity, because it is not our peers but the public, represented by politicians, that is funding our work through taxes. By tradition, few scientists are truly equipped—or interested in—communicating outside their chosen silos, and publications in non-scientific magazines are not only undervalued among hard-core scientists and tenure committees, but they are also often considered distractions from “real work.” In truth, it is quite as hard, if not harder, to convey to non-experts what a new scientific endeavor attempts to do than to communicate a technical advance to peers from the same field. The reluctance of practicing scientists to engage in publicity is therefore understandable but must ultimately not be allowed as an excuse.

Informing the public can take many forms. It is indeed possible to write “popular” books on complex scientific questions, if tone and style are adjusted without compromising the truth. But the time must be ripe, as books on topics that are too unfamiliar do not tend to fare well, whereas books published too late may be considered as not providing any new perspectives. Examples of well-written and well-selling books include *Chaos* by James Gleick, (1997) and *Linked: The New Science of Networks* by Albert-László Barabási (Barabási, 2002). We have tried to share systems biology with the public in a similar manner, but with considerably less success (An, 2014; Voit, 2016; Voit, 2020), arguably because of premature timing. Nonetheless, the public must be informed in a positive, exciting fashion, lest general interest in systems biology is likely to fade.

Alternatives to books are interviews on radio or TV and articles in daily newspapers, now supplemented with exposure via podcasts and social media. For instance, it can be effective to use iTunes, Spotify, LinkedIn, or Twitter (X) as a platform for sharing academic successes, although the latter is not necessarily effective for discourse because of its short format. However, new alternatives such as Mastodon (Mastodon, 2023) are beginning to emerge. Other options are short video blogs (“vlogs”) on YouTube, such as Kurzgesagt (2023) and CrashCourse (CrashCourse, 2023). As noted above, podcasts might be effective ways of sharing information. Examples include TWIM (TWIM, 2023), Science for the People (SOP, 2023) or *Nature*’s podcasts (Nature_Podcast s, 2023). These are often in the format of late-night talk shows where the host brings on a guest to talk about her/his recent study or book. If the guest is able to present the new work in an enjoyable fashion that is easy to understand, this mechanism might be the best venue for amplifying the impact of scientific research. It is clear that it takes skill to explain a complex subject and make it enjoyable to the public. While scientists sometimes lack this skill, a class of science communicators is emerging, who specialize in exactly this translation of scientific jargon into easily digestible news. It will be important to inform these communicators well.

Another option may be the organization and hosting of open-access online symposia, for instance, led by *Frontiers in Systems Biology*. This mechanism would target students first but might be extended to the public later. It would bring students interested in systems biology together to foster networking and the exchange of knowledge. While many scientific meetings have symposia dedicated to trainees at various levels, there is currently no established venue for students of systems biology. Moreover, given that many students, especially those in developing nations, simply do not have the means to travel to such meetings, we envision one or a series of online systems biology meetings that would be organized by and aimed at students. The meetings would feature student-led systems and computational biology research in both basic and translational science. At these meetings, senior faculty mentors may be invited to present their perspectives on systems biology and be available to discuss questions and concerns brought up by the students. *Frontiers in Systems Biology* has pioneered a series of Research Topics entitled *Emerging Talents in Systems Biology*, and this conference would be an ideal opportunity to connect to that topic with regard to student-led publications.

## Conclusions and outlook

In this perspective, we have laid out one particular vision of how systems biology might develop in the near future. While there was reason for us to focus on the presented topics, the future is always uncertain, of course. The main cause for predictions to fail is the human nature of extrapolating trends from the recent past toward the future in a smooth monotonic fashion, which by default is linear. In many cases, this underlying assumption is correct, at least qualitatively, and predictions not too far into the future have a good chance of coming true. However, history has made it clear beyond doubt that technology, science, and society develop gradually for some time, while then being dramatically changed by strong discrete perturbations, which one may call singularities (Ulam, 1958), punctuated equilibria (Eldredge et al., 1972) or black swans (Taleb, 2017). These events disrupt gradual trends and put technology, science, and society onto drastically different trajectories. It is quite obvious that these disruptive events are impossible to predict, both in terms of time and even their nature. For systems biology, it could be, for instance, that a truly new math would be developed, as speculated by Leroy Hood (*pers. comm.*) and others. More likely seem to be new experimental techniques that all of the sudden might overcome some of the current obstacles of systems biology, such as extracting sufficient high-quality parameter values from observation data. Another likely suspect could be the increased and refined use of artificial intelligence which could, for instance, be trained to design large mathematical or data-driven models without error, perhaps in the context of Large Language Models (LLMs) such as Generative Pretrained Transformers (GPT). One challenge for the latter would be the choice of appropriate mathematical representations, but this challenge could be overcome, at least as a default, by using so-called nonlinear canonical models that are constructed according to strict rules, which would be perfect for automation, yet are flexible enough to permit capturing all relevant nonlinearities (e.g., (Savageau, 1996; Voit, 2013)). Other “blue-sky” ideas may change the face of systems biology in ways we simply cannot foresee.

Whatever the future may bring, systems biology has emerged from very humble beginnings (Mesarović,2004; Mesarović, 1968) to become a major force of biomedical research, and it will be exciting to experience where it might lead.
